# Math anxiety and science anxiety are associated with spatial cognition and STEM interest in deaf, hard of hearing, and hearing people

**DOI:** 10.1038/s41539-025-00336-z

**Published:** 2025-07-09

**Authors:** Rachel Gabriella Pizzie, Rachel Marie Sortino, Christina Eun-Young Kim, Rachel Inghram

**Affiliations:** 1 Educational Neuroscience Program, Gallaudet University, Washington, DC USA; 2 Clinical Psychology Program, Gallaudet University, Washington, DC USA

**Keywords:** Psychology, Human behaviour, Psychology, Human behaviour

## Abstract

Many students in STEM experience decreased performance due to anxiety, namely math and science anxiety. However, spatial skills are correlated with better STEM outcomes. Our research addressed a previous gap in the literature, investigating if STEM anxiety or spatial experiences have a stronger relationship with STEM outcomes. In this online study, we explored which factors were related to STEM outcomes in a sample of deaf, hard of hearing (DHH), and hearing adults (*N* = 115) who had experience with American Sign Language, which has been associated with improved spatial skills. Participants completed a mental rotation task, and self-reported interest in STEM, and anxiety. Results showed math and science anxiety were significant predictors of mental rotation performance and interest in studying STEM, even when accounting for other spatial factors. For DHH and hearing people alike, math and science anxiety are important factors that must be addressed to encourage STEM success.

## Introduction

As technology and science increasingly play important roles in our daily lives, there is a need for students to pursue careers in Science, Technology, Engineering and Mathematics (STEM). Students in the US are often encouraged to take classes and choose majors and eventual careers that require mathematics and science knowledge and reasoning. Cognitive abilities as well as interest in technical and scientific skills are strong and significant predictors of outcomes like being interested in and choosing a STEM major^1^. Spatial skills, or ways of thinking and approaching information using visualization, navigation, and mental imagery are also associated with better performance in STEM^2–4^. Additionally, cognitive and motivational factors like self-efficacy and self-reported interests in math and science are found to play a significant role in STEM career interest^5,6^. However, there are a number of obstacles for career interest and achievement in STEM fields. In addition to the challenging material taught in STEM classes, many students must also overcome internal challenges like anxiety about math and science content and beliefs and attitudes about STEM success. In addition, many students must contend with systemic barriers including pervasive stereotypes and lack of accessibility.

In order to promote participation and achievement in STEM majors and careers, we must identify factors that support and detract from success, particularly for individuals who have been historically excluded from success in these fields. Only 3% of the STEM workforce reports experiencing at least one disability^7^. One reason for this low participation rate is barriers to access, for example, some students who are d/Deaf, DeafBlind, or hard of hearing (DDBHH) struggle to have full access to content in STEM classes^8^. Here we refer to DDBHH communities, which are inclusive of individuals who experience hearing loss, and acknowledging that these communities could also include DeafDisabled, LateDeafened individuals, and individuals with auditory processing disorders. However, experience with a spatial language, such as American Sign Language (ASL), that students who are DDBHH likely have experience with, may confer benefits in cognitive skills and motivational factors that may be advantageous for pursuing STEM fields^9,10^.

In the present study, we explored STEM-related factors a sample of DDBHH and hearing individuals (*N* = 115), all of whom had beginner to advanced experience with a spatial language: ASL. Our goal was to explore how anxiety, spatial habits, and experience with a spatial language relate to two outcomes that are predictive of better STEM outcomes: performance of spatial skills, and self-reported interest in studying STEM fields^2–4^. Our goal was to understand what factors are the best predictors of these STEM outcomes in DDBHH and signing communities. We explored whether deaf and hard of hearing (DHH; Note: Some studies do not discuss DeafBlind or other d/Deaf+ populations, and therefore we limit the scope of our discussion to DHH people) individuals experience academic anxieties and other deleterious factors more or less than hearing individuals. In particular, we explored these factors within a sample of adults that all have varying degrees of experience with a spatial language (ASL), which we predicted might confer additional expertise with spatial thinking. Whereas much research in the DHH community compares DHH signers with hearing nonsigners, this novel research explores a range of ASL experiences (from beginner to expert) across both DHH and hearing groups. We investigated how different factors such as anxiety about math, science, and spatial skills, spatial habits, visualization skills, and experience with a spatial language are related to STEM outcomes like spatial skills and interest in studying STEM. This study addresses an important gap in the literature, exploring the relative influence of anxiety and spatial thinking on STEM outcomes in a unique sample of people who may have benefitted from learning a spatial language, ASL.

Spatial skills are related to improved academic performance in STEM domains^2^, and provide foundational cognitive skills that lead to success in STEM education, especially in mathematics. Spatial skills are associated with better performance on tests of mathematical performance and ability, including tests of basic numeracy, and specific course-related assessments of algebra, geometry, calculus, and large scale standardized tests, such as the Program for International Student Assessment, PISA^11^. A meta-analysis of the relation between spatial ability and mathematical outcomes found that even when accounting for mediation by reasoning skills, there was a significant and unique relation between spatial skills and mathematical skills^12^. Large-scale longitudinal studies tracking spatial ability suggest that spatial ability at adolescence was an important factor in individuals who went on to study STEM fields, and that educational and occupational outcomes were related to spatial ability^13^.

Spatial thinking and habits are associated with transfer to other improved STEM skills, and are often augmented by spatial teaching practices. For example, in kindergarteners, building block interventions were related to building a variety of spatial skills^14^. Engaging in more spatial activities in childhood was correlated with more spatial activities in adolescence and increased “spatial habits of mind,” or thinking strategies that use spatial skills^15^. In characterizing strategies for solving math problems, a visual-schematic (visualizer) strategy was associated with greater success in solving problems, whereas a pictorial strategy was not^16^. A tendency toward spatial visualization was also related to increased scientific creativity^17^. Using a high school geospatial mapping course as a training paradigm, spatial habits were also increased by taking this geospatial course in high school compared to students enrolled in other courses of study^18,19^. Spatial habits are an indicator of applied spatial skills in and outside of the classroom.

Spatial training suggests spatial skills are malleable, and is also correlated with increased STEM skills, especially mathematics. In elementary school children, spatial activity training was related to improved number line knowledge^20^. In six to eight-year-olds a mental rotation training intervention was associated with improved calculation scores compared to a crossword puzzle training intervention^21^. A 32-week-long teacher-led spatial intervention was related to improvements in young children’s spatial skills and spatial number skills compared to a business-as-usual control^22^. But young children are not the only students to benefit from improved spatial training. In high school-aged students, a spatial intervention in the form of a spatially-focused pedagogical framework was related to gains in spatial skills and mathematics performance after the intervention^23^. Students showed improvement in tests of numbers, algebra, and geometry compared to the control group who received no spatial training. Even beyond mathematics, compared to students who took similar non-spatially-oriented classes, students who took a spatial mapping class in high school showed improvement in reasoning skills and showed changes in neural activity in regions of the brain associated with spatial ability^19^. These studies demonstrate that spatial training, interventions, and activities are related to improving not only spatial skills, but math and science skills that are important for STEM achievement.

Interest in studying STEM is also an important factor in STEM achievement, and past research has explored how interest in STEM compares to other factors for predicting STEM achievement, such as performance and ability in STEM classes. Past success in STEM classes was related to interest in future studying, though it seems to be field-specific^5^. For example, if a student has had past success in science classes, they are more likely to want to pursue a career in science, but not necessarily a career in math or engineering^5^. Although, a longitudinal study found that while self-interest in STEM topics was related to later pursuit of STEM majors, it was not as influential as self-perceived STEM ability^6^. Whether or not students perceived themselves as “good at” STEM had more of an influence on their decision to major in STEM fields than their actual “interest in” STEM^6^. However, a study that looked at data from over 75,000 students exploring STEM interest on graduation outcomes found that STEM interest played an important role^24^. This large-scale study found that after controlling for academic preparation (taking advanced STEM coursework and ACT scores), students who both expressed an interest in STEM and stated an intent to major in STEM were more likely to graduate with a STEM degree than students who showed no interest in STEM. Moreover, students who expressed both an interest in STEM and intent to major were more likely to graduate with a STEM degree than students who expressed either an interest or an intended major^24^. Whereas interest in STEM is not enough to guarantee success in STEM fields, it is an important factor to consider when trying to increase participation in STEM.

DHH students pursue STEM majors at approximately the same rate as their hearing peers, in fact a higher percentage of DHH students graduate with associate degrees in STEM as compared to their hearing counterparts^25^. However, a significantly lower percentage DHH students receive bachelor’s degrees in STEM as compared to hearing students, resulting in DHH students having fewer opportunities for career advancement within the field^25^. The fact that DHH students sign up to major in STEM at similar rates as hearing peers indicates that they have similar levels of interest in studying STEM, but may face additional barriers in pursing higher level degrees in STEM fields^26^. For example, DHH students who are interested in STEM often struggle to find qualified interpreters with sufficient STEM content knowledge, especially for advanced STEM courses, leading to retention problems despite the students’ interest in STEM^8^.

Considering the difficult nature of success and achievement in STEM education, DDBHH communities also face additional challenges in STEM fields and often lag behind in achievement compared to their hearing peers^8,27,28^. DDBHH students often encounter barriers in accessibility, where information is often presented via lecture with few examples of visually accessible material, or the expertise of ASL-English interpretation may impact student understanding of difficult STEM concepts. In tandem with lack of accessibility, ASL also lacks a broadly accepted lexicon of science-related signs^29^. Often DDBHH students must rely on numerous fingerspelled terms and English-based translations to understand many of the complex concepts in STEM classes.

However, ASL is well-suited to convey the complex visuospatial information and concepts related to numeracy, magnitude, and spatial relationships in STEM fields, much in the same way that high-level conceptual gestures also augment understanding of scientific concepts^27,28,30–32^. Providing spatial practice through language could augment spatial skills in a method that is well-suited to STEM training^9,33^. ASL incorporates rich spatial information to convey concepts, and offers a unique pathway to enhance spatial cognition and spatial skills. Thus, experience and training with a spatial language could also confer benefits to spatial skills, and by extension, support STEM success.

There is some debate as to whether some DHH individuals have an advantage in spatial skills, and whether this is due to reliance on visual information due to lack of access to sound, or attributed to training with visuospatial language like ASL. A variety of studies have investigated whether DHH individuals show an inherent advantage for spatial skills or learning spatially, with mixed results^34–36^. Some studies with DHH participants show that DHH people show a predisposition for visual information through increased visual attention, and perhaps a widening of visual attention span^36^. Hearing loss does not necessarily seem to confer advantages for spatial skills, but access to and expertise with spatial language does seem to be associated with spatial skill improvement^9,34,35^. Spatial language like ASL may serve as a kind of training for spatial skills associated with language use^10,37^. In this study, we will investigate whether spatial skills, measured on a mental (MR) rotation task, are correlated with self-reported sign language experience and expertise in adult signers who are DHH and hearing. Whereas the embodiment of gesture shows some advantages for spatial thinking^32,38,39^, we hypothesize that experience with ASL as a visuospatial language regardless of hearing status will be correlated with advantages for spatial skills like error rate on the MR task, and improvement in other factors related to STEM success, like interest in STEM. In the present study, we will explore these factors in both DHH and hearing people that have minimal to native/expert experience with ASL in order to investigate the potential influence of spatial language on spatial skills.

Although there are factors that encourage success in STEM fields, there are also challenges that detract from STEM achievement, such as academic anxiety. Individuals from underrepresented and historically excluded backgrounds in STEM, such as those with disabilities, may be more likely to experience academic anxiety; for example, some students from low socioeconomic status backgrounds are more likely to experience math anxiety^40–45^. Academic anxiety refers to negative emotions, anxiety, and avoidance of specific domains of academic information, such as mathematics, science, and spatial skills. Importantly, different kinds of academic anxiety not only result in negative emotion and aversive experiences, but also have behavioral consequences^46^. For example, math anxiety is associated with decreased performance in math^42,43,47,48^. Spatial anxiety is correlated with decreased performance on spatial skills^45,48^; science anxiety is associated with decreased performance on specific science domains, like chemistry^49^. Over time, these academic anxieties detract from school performance, creating a vicious cycle where anxious students avoid engaging with the material and learn less, which leads to more anxiety and further underperformance and avoidance^43,46,50^.

Thus far, there is limited research on academic anxiety in DHH people. Existing studies have primarily focused on math anxiety and have yielded mixed findings^51,52^. The results of one study in Iranian female students indicated that DHH participants (*n* = 63) self-reported more math test anxiety than hearing female peers^51^. Moreover, mathematics self-efficacy was lower in DHH than hearing students. DHH students also showed decreased mathematics performance in school compared to the hearing group. The results of this study acknowledged the importance of language access for DHH students, but did not account for students’ language of instruction or other language-related factors in their educational background. This study only included female students, and more work should explore whether this group difference remains when both genders are included in the sample. Another study conducted in a large sample of US students (*N* = 296) examined how a number of different factors were related to math anxiety in DHH and hearing students^52^. When controlling for general (trait) anxiety, math anxiety in DHH students was significantly related to mathematics feelings and school environment, and was not related to age, gender, and parental behaviors. DHH students who reported elevated math anxiety also reported higher ratings of their mathematical skills, math importance, school emphasis on STEM and school support and resources. Positive ratings of the school environment and positive feelings toward mathematics were positively associated with math anxiety in DHH students, which was an unexpected result. The authors suggested this result might be attributed to DHH individuals’ underrepresentation in science, indicating that math anxiety may be positively associated with positive value of math and might be reflective of a perceived high risk of failure even when DHH students want to succeed in STEM. Positive feelings toward mathematics, the school environment, and parental behaviors showed different relationships with math anxiety in the DHH and hearing groups. Across both DHH and hearing groups in this study, math anxiety was not associated with likelihood of studying a STEM field. Although this study reported an overall difference in math anxiety that showed elevated math anxiety in DHH students compared to hearing students, the factors between DHH and hearing groups can be attributed to varying attitudes, values, and educational environments. Considering the incredible diversity of language backgrounds and educational experiences of DHH individuals, it is vital to consider how a variety of educational experiences may contribute to math anxiety or other STEM outcomes.

In the current study, we explored group comparisons between math anxiety, science anxiety, and spatial anxiety in DHH and hearing individuals. We predicted that due to systemic barriers in STEM, our DHH group would report slightly higher math and science anxiety than their hearing peers. However, because practice with spatial language may ameliorate spatial anxiety, we did not hypothesize that the DHH group would report increased spatial anxiety compared to the hearing group of participants, especially because all participants in the study had at least some minimal experience with ASL. This study adds to the literature exploring whether DHH individuals experience increased academic anxieties, whether this results in robust differences in math, science, and spatial anxiety, and whether this also relates to outcomes like spatial skills and interest in studying STEM.

As math, science, and spatial anxiety are likely all related to experiences with math and science learning, past research has explored the overlap in these domains, especially math anxiety and spatial anxiety^48^. In one study, spatial anxiety, general anxiety, and small-scale spatial skill were all related to math anxiety^53^. An additional study explored the domain specificity of math and spatial anxiety together, testing the specificity of each of these anxieties on math and spatial tasks^48^. Math anxiety and spatial anxiety predicted domain-specific performance on math and spatial skills, respectively. When measures of math, spatial, and trait anxiety were combined to explore differences in task performance, math anxiety accounted for the most variance in both the math task and spatial task performance. These results suggest that math anxiety accounts for variability in math performance and spatial performance, even above and beyond the variance accounted for by spatial anxiety.

In the present study, we explored similar questions as to whether math, spatial, and science anxiety would be related to spatial skills, investigating the overlap between these domains on a mental rotation task. In particular, our sample adds additional interest to these questions by examining these factors in individuals who have varying experience with spatial language, ASL, which gives users additional practice utilizing spatial skills associated with language in DHH and hearing individuals. In our study, we hypothesized that all three domains of math, spatial, and science anxiety would be related to one another. When measured in zero-order correlations, we predicted that all three domains would be related to mental rotation performance, such that increased anxiety would be associated with poorer performance and increased errors. We hypothesized that spatial anxiety would account for the most variance in mental rotation task performance. Although, given the previous results reported in Daker, et al. ^48^, we also considered that math anxiety might account for more variance in mental rotation performance. In examining these relations in a sample of participants that may have additional experience with spatial skills, we also sought to explore the relative contribution of spatial anxiety, math anxiety, and science anxiety compared to self-reported spatial language experience, and how these all relate to mental rotation performance. Understanding the potential contributions of spatial language experience and anxiety in mental rotation skill is a novel contribution of this study to the body of literature on spatial cognition and STEM outcomes.

In the present study, we wanted to explore factors related to two common predictors of STEM outcomes: spatial skills and interest in studying STEM fields. In particular, we investigated some common factors that detract from STEM outcomes, such as math anxiety, science anxiety, and spatial anxiety. We also investigated some common factors that support STEM development, such as spatial habits. In this study, we had the opportunity to explore the relations of these factors within the DHH community, investigating whether experience with signed language was associated with advantages for cognitive skills and motivation related to STEM success in DHH and hearing individuals. Our aim was to replicate and extend some previous research on these topics, while investigating these factors in a sample of DHH and hearing individuals, exploring their combined effect on outcomes related to STEM success. This novel research explored group differences between DHH and hearing samples, characterizing academic anxieties, spatial habits, spatial skills and interest in STEM in an understudied community. This research also represents a unique exploration into how experience with a visuospatial language, ASL, is related to academic anxieties, spatial skills, spatial habits, and interest in STEM.

1. *RQ1: Do academic anxieties, spatial skills, and STEM interest differ between DHH individuals and hearing individuals?*

- Do DHH participants experience more math anxiety, science anxiety, and spatial anxiety than their hearing peers?
- Do DHH participants evidence better spatial habits than their hearing peers?
- Do DHH participants evidence better performance on spatial skills than their hearing peers?
- Do DHH participants evidence more interest in STEM compared to their hearing peers?

2. *RQ2: Are there associations between academic anxieties, spatial skills and habits, and spatial language experience in a sample with experience with ASL, regardless of hearing status?*

- Are spatial skills and spatial habits each positively associated with spatial language experience?
- Are math anxiety, science anxiety, and spatial anxiety associated with one another?
- Are math anxiety, science anxiety, and spatial anxiety associated with spatial habits?
- Are spatial habits associated with better spatial skills?
- Are spatial habits associated with interest in studying STEM fields?
- Are math anxiety, science anxiety, and spatial anxiety associated with spatial skills?
- Are math anxiety, science anxiety, and spatial anxiety associated with interest in studying STEM fields?

3. *RQ3. Do academic anxieties, spatial habits, experience with ASL, or hearing status uniquely predict performance on spatial skills?*
4. *RQ4. Do academic anxieties, spatial habits, experience with ASL, or hearing status uniquely predict interest in STEM?*

## Results

### Analysis plan

To address Research Question (RQ) 1, we first conducted *t*-tests to evaluate any group differences between the deaf/hard of hearing (DHH) and hearing groups. In order to accommodate our DHH and hearing groups of unequal size and potentially unequal variances we utilized *t*-tests with the Welch (Satterthwaite) method to correct the degrees of freedom to estimate statistical significance of group differences. To address RQ 2, we then evaluated zero-order correlations and relations between variables of interest. Statistical analyses were completed in R^54^. We wanted to look at the relationship between each factor independently. We utilized Pearson correlations and *ɑ* = 0.05 to evaluate relations between questionnaires. *Z*-tests were used to compare correlation strength where applicable. To address RQ 3 and 4, we explored which factor would be associated with the most variance in predicting mental rotation error rates and self-reported STEM interest. Using a linear regression (method = “Enter”), we sought to evaluate which predictor variables might account for the most variance in spatial skill performance using commission error rate on the mental rotation task as our primary outcome of interest. We utilized the same method to evaluate which predictor variables would be related to interest in studying STEM. Additional analyses including gender are included in the Supplementary Material (Supplementary Results 1, Supplementary Tables 1–3). Analyses utilizing a mental rotation accuracy measure instead of mental rotation commission error rate are also included in the Supplementary Material (Supplementary Results 2, Supplementary Tables 4–6). Additional analyses considering RQ3 and RQ4 for DHH and Hearing groups separately are included in the Supplementary Material (Supplementary Results 3, Supplementary Tables 7–10).

**RQ1: Do academic anxieties, spatial skills, and STEM interest differ between DHH individuals and hearing individuals?**

In this first question, we used an approach typical of developmental science, characterizing and describing the differences between DHH and hearing participants in our sample.

**RQ1A: Do DHH participants experience more math anxiety, science anxiety, and spatial anxiety than their hearing peers?**

We hypothesized that DHH people would report increased math and science anxiety, and would not report increased spatial anxiety compared to hearing peers due to experience with spatial language. However, this hypothesis was only partially supported (Table 1), such that DHH participants reported increased science anxiety (*M*_*DHH*_ = 0.11) compared to hearing participants (*M*_*H*_ = −0.26). For math anxiety and spatial anxiety, there were no significant differences in how DHH and hearing participants reported their levels of anxiety, *p*’s > 0.05.

**Table 1:** Zero-order correlations between academic anxiety, spatial habits and skills, ASL experience, STEM interest, and differences in hearing status

	Math Anxiety	Science Anxiety	Spatial Anxiety	Spatial Habits	Visualizer	ASL-Experience	MR Comm. Error Rate	STEM Interest
Math Anxiety	--	0.25*	0.26*	−0.42***	0.16	0.00	0.32**	−0.53***
Science Anxiety	0.26**	--	0.30**	−0.30**	0.30**	−0.13	0.28*	−0.36**
Spatial Anxiety	0.28**	0.29**	--	−0.09	0.16	0.05	0.14	−0.32**
Spatial Habits	−0.44***	−0.31***	−0.21*	--	−0.34**	0.00	−0.24*	0.42***
Visualizer	0.19*	0.27**	0.18	−0.40***	--	−0.17	0.10	−0.14
ASL-Experience	0.12	0.05	0.06	−0.05	−0.08	--	−0.13	−0.07
MR Commission Error Rate	0.39***	0.34***	0.22*	−0.26**	0.13	0.08	--	−0.19
STEM Interest	−0.61***	−0.37***	−0.33***	0.41***	−0.16	−0.19*	−0.25**	--
Hearing Status	*t*(56.67) = 0.68, *p* = 0.50 NS	*t*(75.18) = 2.02, *p* = 0.04* DHH > H	*t*(56.68) = 1.11, *p* = 0.27 NS	*t*(61.48) = −1.19, *p* = 0.23 NS	*t*(67.51) = 0.07, *p* = 0.94 NS	*t*(41.62) = 5.74, *p* < 0.001*** DHH > H	*t*(69.52) = 1.31, *p* = 0.20 NS	*t*(59.74) = −1.30, *p* = 0.20 NS

Correlations below the diagonal represent relations in all participants (*N* = 115). Correlations above the diagonal were calculated in DHH participants only (*N* = 81). For specific questionnaire names for each construct please see Table 4. Pearson correlations and *t*-tests for hearing status were calculated with alpha = 0.05. Statistically significant effects for differences in hearing status are discussed in-text.

*DHH* deaf and hard of hearing, *H* Hearing, *NS* no significant group differences.

*p < 0.05, **p < 0.01, ***p < 0.001.

**RQ1B: Do DHH participants evidence better spatial habits than their hearing peers?**

Here we compared self-reported spatial habits using the SHOMI-VIS subscale, and whether participants reported tendencies to be a “visualizer,” on the VVQ-VIS subscale (Table 4). Across both measures, DHH participants and hearing participants did not differ in their self-reported spatial habits, all *p*’s > 0.05 (Table 1).

**RQ1C: Do DHH participants evidence better performance on spatial skills than their hearing peers?**

We predicted we would replicate previous research suggesting that DHH signing individuals would have lower commission error rates on the MR task. However, our results showed that there were no differences in MR commission error rate based on hearing status. The DHH group did not perform better on this task compared to the hearing group (Table 1), *p* > 0.05.

**RQ1D: Do DHH participants evidence more interest in STEM compared to their hearing peers?**

Here we compared DHH and hearing participants on their self-reported interest in studying STEM fields. Our results (Table 1) showed no difference between groups in self-reported interest in studying STEM fields, *p* > 0.05.

**RQ2: Are there associations between academic anxieties, spatial skills and habits, and spatial language experience in a sample with experience with ASL, regardless of hearing status?**

Whereas our first research question explored group differences, this research question will now explore the associations between ASL language experience, academic anxieties, and spatial habits and skills across the whole sample.

**RQ2A: Are spatial skills and spatial habits each positively associated with spatial language experience?**

In addition to exploring the effects of hearing status, we predicted that spatial language experience (ASL-Summary), would be correlated with improved performance on the mental rotation task. From our results (Table 1), more experience with ASL was not associated with decreased commission error rates on the MR Task, *p* > 0.05. Similarly, more ASL experience was not related to differences in spatial habits or visuospatial tendencies. ASL experience was also not correlated with math, science, or spatial anxiety. Contrary to our hypotheses, our results did not support the idea that more experience and expertise with spatial skills through a spatial language (ASL) would confer benefits for spatial thinking or skills, and was not related to anxiety about math, science or spatial content.

**RQ2B: Are math anxiety, science anxiety, and spatial anxiety associated with one another?**

We hypothesized that math, science, and spatial anxiety would all be significantly related to one another. Indeed, our hypothesis was confirmed, and math, science, and spatial anxiety were positively related to one another (Table 1) with relations between *r* = 0.25–0.30 in the full sample. As all of these questionnaires assess anxiety about similar or overlapping cognitive skills, it is not surprising that anxiety in one domain would be related to the others.

**RQ2C: Are math anxiety, science anxiety, and spatial anxiety associated with spatial habits?**

We also wanted to explore whether spatial habits and visualizer tendencies were correlated with academic anxiety, predicting that increased anxiety would be related to decreased spatial habits or preferences for visual information. Indeed, we found that spatial habits were inversely correlated with math, science, and spatial anxiety (Table 1). Results were mixed for the tendency to be a “visualizer.” Overall, these results show that using spatial habits or visuospatial tendencies are inversely related to math, science, and spatial anxieties.

**RQ2D: Are spatial habits associated with better spatial skills?**

We predicted that increased spatial habits and visualization would be associated with better performance on a mental rotation task. We found that spatial habits, especially those associated with visualization, were positively correlated with decreased MR commission error rate (Table 1, *r* = −0.26). However, tendency to be a “visualizer,” or a person who often uses visuospatial tendencies and skills to represent information, was not related to MR commission error rate, *p* > 0.05.

**RQ2E: Are spatial habits associated with interest in studying STEM fields?**

We predicted that, like spatial skills, increased self-reported spatial habits and visualization would be related to more interest in studying STEM. As predicted, self-reported spatial habits on the SHOMI were correlated with increased interest in studying STEM (Table 1, *r* = 0.41). However, self-reported “visualizer” tendency was not associated with interest in studying STEM, *p* > 0.05.

**RQ2F: Are math anxiety, science anxiety, and spatial anxiety associated with spatial skills?**

We hypothesized that all three types of anxiety would be related to commission error rate on a MR task, such that increased anxiety would be correlated with poorer performance and increased commission errors. We further hypothesized that spatial anxiety (mental manipulation) would account for the most variance, or have the strongest relation with MR commission error rate, because of the specificity of this domain. However, we found that math anxiety had the strongest relation with MR commission error rate (Table 1), though statistical comparisons to other types of anxiety indicated that this correlation was not significantly stronger, *p* > 0.05. Our data confirmed our predictions that math, science, and spatial anxiety are related to MR commission error rate (Table 1), and that more anxiety was related to poorer performance on the MR task.

**RQ2G: Are math anxiety, science anxiety, and spatial anxiety associated with interest in studying STEM fields?**

We hypothesized that increased anxiety would be related to decreased interest in STEM. All three types of anxiety are negatively related to interest in studying STEM (Table 1). As we observed in the relations between anxiety and MR commission error rate, math anxiety was most strongly related to STEM interest (*r* = −0.61), and this relation was almost twice the size of the correlations between STEM interest and science anxiety (*r* = −0.37, *Z*(115) = 2.40, *p* = 0.01) and spatial anxiety (*r* = −0.33, *Z*(115) = 2.74, *p* = 0.006).

**RQ3: Do academic anxieties, spatial habits, experience with ASL, or hearing status uniquely predict performance on spatial skills?**

In this analysis, we sought to evaluate which factors, including anxieties, spatial habits, experience with ASL, and hearing status would account for the most variability in spatial skills performance (measured by the commission error rate on the MR task), accounting for all these variables (and their covariance) at once. In this linear regression, MR commission error rate was the main outcome variable, while we identified the following independent variables and entered them into the linear regression model: spatial anxiety, math anxiety, science anxiety, spatial habits of mind (visualization subscale), tendency to be a visualizer (VVQ-VIS), ASL Experience, and hearing status. All continuous self-reported questionnaire measures were Z-scored in the model.

The results of this regression model show that math anxiety and science anxiety are significant positive predictors of increased commission error rates on the mental rotation task (Table 2). As math anxiety and science anxiety increased, participants made more incorrect responses on the mental rotation task. Surprisingly, spatial anxiety did not account for unique variance (*p* > 0.05) when math and science anxiety were included in the same model, indicating that these variables are strong predictors above and beyond the variance accounted for by spatial anxiety. Similarly, we did not observe that self-reported habits such as visualization were significantly related to mental rotation commission error rate. Finally, we did not observe that experience with a spatial language (ASL) or hearing status was related to mental rotation commission error rate. Overall, we find that math and science anxiety accounted for the most variance in spatial skills, over and above other variables related to spatial thinking.

**Table 2:** Regression results using mental rotation commission error rate as the criterion

Predictor	*b*	*b* 95% CI [LL, UL]	*sr*^*2*^	*sr*^*2*^ 95% CI [LL, UL]
(Intercept)	32.78**	[29.62, 35.94]		
Spatial Anxiety	0.84	[−1.83, 3.50]	0.00	[−0.01, 0.02]
Math Anxiety	4.31**	[1.47, 7.16]	0.07	[−0.02, 0.15]
Science Anxiety	3.31*	[0.53, 6.10]	0.04	[−0.02, 0.11]
Spatial Habits	−0.75	[−3.75, 2.24]	0.00	[−0.01, 0.02]
Visualizer	−0.39	[−3.13, 2.35]	0.00	[−0.01, 0.01]
ASL-Experience	−0.14	[−3.58, 3.29]	0.00	[−0.00, 0.00]
Hearing Status	−0.93	[−7.56, 5.69]	0.00	[−0.01, 0.01]

Overall Fit: *R*^*2*^ = 0.221**, 95% CI [0.05,0.31].

Spatial Anxiety, Math Anxiety, Science Anxiety, Spatial Habits, and the Visualizer subscale were all Z-scored for this analysis. For questionnaire descriptions please see Table 4. A significant *b*-weight indicates the semi-partial correlation is also significant. *b* represents unstandardized regression weights (though, on the *Z*-scored variables, these represent standardized scores). *sr*^*2*^ represents the semi-partial correlation squared. *LL* and *UL* indicate the lower and upper limits of a confidence interval, respectively. VIF for each variable was <1.5 indicating low multicollinearity between variables.

*p < 0.05, **p < 0.01.

**RQ4: Do academic anxieties, spatial habits, experience with ASL, or hearing status uniquely predict interest in STEM?**

Similar to our predictions for spatial skills, we sought to explore what factors would predict the most variance in self-reported interest in studying STEM fields. Again, we used linear regression with STEM interest as our outcome variable, with spatial anxiety, math anxiety, science anxiety, spatial habits of mind, visualization tendency, ASL Experience, and hearing status as independent variables. In addition, in this model we included spatial skills (MR commission error rate) as an independent predictor variable, exploring whether we would replicate previous research suggesting that spatial skill performance predicts interest in studying STEM fields.

The results of this linear regression show that math anxiety and science anxiety are significant negative predictors of self-reported interest in studying STEM fields (Table 3). As anxiety increases, participants’ self-reported interest in studying STEM decreased significantly. As with our model for MR commission error rate, math and science anxiety were significant predictors above and beyond other spatial factors like spatial anxiety, spatial habits, and ASL experience and hearing status, all *p*’s > 0.05. Our variables examining spatial language experience and whether participants were DHH or hearing did not account for significant variance in STEM interest. Interestingly, in this model we also included MR commission error rate as a predictor variable, but it was not a significant predictor of interest in studying STEM. These results suggest that anxiety about math and science are statistically significant and important predictors of interest in STEM, even above and beyond other spatial variables including spatial skill performance. Taken together, the results from both linear regressions suggest that math anxiety and science anxiety are robust predictors of spatial skills and interest in studying STEM.

**Table 3:** Regression results using STEM Interest as the criterion

Predictor	*b*	*b* 95% CI [LL, UL]	*sr*^*2*^	*sr*^*2*^ 95% CI [LL, UL]
(Intercept)	2.04**	[1.64, 2.44]		
Spatial Anxiety	−0.12	[−0.27, 0.03]	0.01	[−0.02, 0.05]
Math Anxiety	−0.45**	[−0.62, −0.29]	0.16	[0.05, 0.26]
Science Anxiety	−0.20*	[−0.36, −0.04]	0.03	[−0.02, 0.08]
Spatial Habits	0.13	[−0.03, 0.30]	0.01	[−0.02, 0.05]
Visualizer	0.04	[−0.11, 0.19]	0.00	[−0.01, 0.01]
ASL-Experience	−0.13	[−0.32, 0.06]	0.01	[−0.02, 0.04]
Hearing Status	−0.05	[−0.42, 0.32]	0.00	[−0.01, 0.01]
Mental Rotation Comm. Error Rate	0.00	[−0.01, 0.02]	0.00	[−0.01, 0.02]

Fit: *R*^*2*^ = 0.461**, 95% CI[0.27,0.54].

Spatial Anxiety, Math Anxiety, Science Anxiety, Spatial Habits, and the Visualizer subscale were all Z-scored for this analysis. Questionnaire names are provided in Table 4. A significant *b*-weight indicates the semi-partial correlation is also significant. *b* represents unstandardized regression weights (though, on the *Z*-scored variables, these represent standardized scores). *sr*^*2*^ represents the semi-partial correlation squared. *LL* and *UL* indicate the lower and upper limits of a confidence interval, respectively. VIF for each variable was <1.5 indicating low multicollinearity between variables.

*p < 0.05, **p < 0.01.

## Discussion

In this study, we aimed to explore which factors would be important predictors of two important STEM outcomes: spatial skills and interest in studying STEM (RQ3 and 4). In this online study, we investigated several factors that we predicted would be related to these outcomes: anxiety about math, science, and spatial skills, spatial habits, spatial thinking, experience with a spatial language (ASL) and hearing status. We sought to investigate associations between these factors (RQ2), and compare these factors between DHH and hearing groups (RQ1). In particular, this online study examined these factors within signing communities, looking at these variables among people who had varying levels of exposure to ASL including people who identified as deaf, hard of hearing, and hearing. Whereas previous research has investigated academic anxiety, spatial habits and thinking, spatial skills and STEM interest separately, here we were also able to test all these elements within a sample of people that regularly utilize spatial skills in ASL. Our results showed that when accounting for all these variables together, math anxiety and science anxiety accounted for the most variance in commission error rate on a mental rotation task. Similarly, when we explored interest in studying STEM fields, math and science anxiety were also significant predictors of decreased interest in studying STEM, even when accounting for additional variables like self-reported spatial habits, experience with ASL, and hearing status. These results suggest that across both spatial skill performance and STEM motivation, anxiety about math and science detract from success and achievement.

To measure spatial skills, we used a standard mental rotation paradigm, exploring which factors would be related to increased or decreased MR commission error rates. Across both our zero-order correlations, exploring independent relations with MR commission error rate, and the multiple regressions, accounting for all predictors at once, math anxiety and science anxiety were robust predictors of MR commission error rate. As both math anxiety and science anxiety increased, MR commission error rate increased. We observed a statistically significant correlation in the zero-order correlations with spatial anxiety and MR commission error rate. However, when all types of anxiety were included in the multiple regression, spatial anxiety was not a significant predictor of MR commission error rate. This is aligned with other previous research on spatial anxiety^48^, suggesting that math anxiety is a more robust predictor of spatial skills. Although we would have predicted that the domain specificity of spatial anxiety would have resulted in a more robust relation between spatial anxiety and performance of spatial skills, the close interrelation between spatial skills and academic domains like math and science help to explain this result.

We also explored another important facet in STEM success: interest in studying STEM. This motivational factor is key for long-term STEM success, including choosing college majors and careers in STEM^1,5,6^. Like our results for spatial skills, when accounting for all factors together, math anxiety and science anxiety were statistically significant predictors of decreased interest in studying STEM. When accounting for all variables together, only math and science anxiety emerged as significant negative predictors of interest in STEM, which was similar to our result for MR commission error rate. This is especially interesting when considering that MR commission error rate was also included as a predictor in this model. Our results suggest that even above and beyond an actual measure of spatial skill, math and science anxiety accounted for more variance in participants’ interest in studying STEM fields. This is again consistent with previous work suggesting that anxiety is more predictive of academic success in college than measures of math ability^55^.

In addition to our primary results, we sought to explore several research questions that would replicate and extend previous research. First, we explored some previous findings, replicating previous work on academic anxieties: academic anxieties are related to one another, and related to negative performance and motivation in STEM. Our results supported previous research findings^45,47,56,57^, suggesting relations between math, science, and spatial anxieties, such that these factors share some variance (*r’*s = 0.24–0.30), but likely still represent distinct domains. Also replicating previous research, all math, science, and spatial anxiety measures were independently related to spatial skills (MR commission error rate), and STEM interest. This result is consistent with previous research suggesting that anxiety detracts from spatial skill performance and longer-term predictors like interest and motivation for STEM.

For spatial habits of mind^18,19^ and visualization^58,59^, we also explored if these self-reported habits and skills would be correlated with MR commission error rate. As has been shown with previous research, we expected that increased spatial habits would also be associated with better performance on MR commission error rates. Our results supported this conclusion for the Spatial Habits of Mind Inventory (SHOMI-VIS), which was negatively related to increased MR commission error rates. However, self-reported visualization tendencies (VVQ-Vis) were not significantly correlated with better MR performance. These results replicate previous work suggesting that self-reported habitual use of spatial strategies are associated with better performance of spatial skills and activities, but were not related to self-reported tendency to be a “visualizer”^15,18,19^.

To replicate and extend previous research on DHH individuals, we also explored how DHH people might differ from their hearing peers in anxiety, spatial habits, spatial skills and interest in STEM. Past research has explored how DHH and hearing students might differ in levels of math anxiety^51,52^, although the results from previous studies have been mixed, especially in exploring associations between math anxiety and other educational factors in DHH samples. In our results, DHH and hearing groups did not differ in their self-reported average levels of math anxiety or spatial anxiety. DHH individuals reported increased science anxiety compared to their hearing peers. DHH and hearing groups did not differ on spatial habits or tendencies to be a “visualizer.” In addition, we also sought to replicate previous research suggesting that DHH individuals show an advantage on spatial skills^9,34–36^. In our study, the DHH and hearing groups of participants did not perform differently on MR commission error rates. As a result, we did not replicate previous research suggesting an advantage for spatial skills or visuospatial attention for DHH compared to hearing individuals. In terms of replicating previous research with DHH people, our results suggest that DHH individuals did not robustly show increased anxiety about STEM fields, and did not show increased performance on spatial skills. Further research will need to explore whether additional moderators affect how anxiety and spatial skill development differentially impact DHH individuals.

We also explored whether exposure to a visuospatial language, ASL, would be associated with reduced anxiety, or increased spatial skills and habits. We posited that increased practice with spatial skills through ASL might confer better skills and would reduce anxiety though more exposure to spatial concepts in everyday practice. This study recruited participants who all had at least some minimal exposure and education in ASL. However, our results did not confirm our hypotheses. Increased ASL experience was not correlated with any of our measures of math, science, and spatial anxiety. ASL experience was not associated with spatial habits of mind or visualization tendencies. ASL experience was not associated with improved spatial skill performance, which did not replicate previous research^9^. However, Kubicek and Quandt also utilized a validated measure of ASL comprehension, whereas the current study used a self-reported ASL summary measure combining ASL comfort, preferences, and age of ASL acquisition. Although our ASL-Summary variable included self-reports of multiple facets of language experiences, including measures of both receptive and expressive skills, our ASL-Summary measure also did not show many robust associations with other measures of spatial cognition. Our results did not suggest that ASL experience was related to better spatial skills, better spatial habits, and was not related to anxiety about math, science, or spatial content. It may be that overall receptive ASL proficiency, as measured by Kubicek and Quandt^9^ is more closely related to MR skills. Overall, although our study sought to explore the influence of spatial language experience, our results suggest that the effects of math anxiety and science anxiety are comparatively more impactful than the effects of language on the STEM outcomes that we measured.

Overall, our results also suggested that our DHH and hearing groups did not significantly or robustly differ from one another on many of our key measures. Again, these results suggest that math anxiety and science anxiety are important factors to consider in STEM outcomes for DHH and hearing individuals alike. We find that in addition to replicating some previous research on academic anxieties and spatial habits, much of our research did not replicate previous research, and did not confirm our hypotheses about the impact of spatial language. Math anxiety and science anxiety were both shown to be important, robust predictors of spatial skills and interest in studying STEM, which are both key factors in overall STEM success.

The current study has some important limitations that must be considered in light of our results. First, this dataset was all collected online and participants likely had heterogeneous experiences completing the study questionnaires and activities compared to a traditional in-lab experiment. Although the research team tried to mitigate some of these factors with additional instructions provided to participants and breaks, the nature of online data collection should be considered in interpreting our results. The measures in this study were designed to present a more coarse-grained picture of spatial skills and interest in studying STEM. For example, for our MR task, participants were presented with stimuli for seven seconds, and given the same amount of time to respond. The timing on this task could have been slightly impacted by internet speed or display, and we were not able to record reliable reaction times. As a result, our main outcome measure for this task was limited to the error rate on this speeded task, which could have made the task more difficult for our participants. Moreover, the MR task only tested one facet of spatial skills, and future studies could test more varied skills. In addition, although we designed an arithmetic task to be presented as a part of this study, due to an error in the task coding, the behavioral data collected were not usable for analysis. Additional research in the future will be able to explore the effects of our variables of interest on math performance in addition to spatial skills performance and interest in STEM.

The present study represents one cross-sectional study of both DHH and hearing signing individuals, but DDBHH communities are incredibly diverse. DDBHH communities represent a great deal of linguistic and educational diversity, and more research is needed to draw conclusions about the nature of the relationships explored in this research. Many of the research questions that we explored in order to replicate previous research did not show the same results as what had previously been found. These differences could be due to differences in measurement (for example, having a measurement of ASL comprehension versus self-reported language experience), or differences in the sample tested. For example, all participants in the present study had at least a minimum amount of beginner experience with ASL. Some previous studies that demonstrated differences in spatial skills and visual attention often contrast deaf signers with hearing nonsigners to explore the differences between these groups. Because all participants in our study had some signing experience (and therefore potentially more spatial skill experience), this could have reduced the differences observed between DHH and hearing groups in previous studies. In addition, this particular study was optimized to collect a range of signing experience, but was not necessarily optimized to dichotomize DHH and hearing groups, and regrettably, because we were only able to collect data from a small sample of DeafBlind participants, we could not draw generalizable conclusions from their data. Our recruitment resulted in a predominance of DHH individuals in our sample, and we did not have equal distribution across both DHH and hearing groups. In addition, we did not have equal distributions of signing skills across both groups, with the DHH group reporting increased scores on the ASL summary measure compared to the hearing group. More research is needed to disambiguate how spatial language interacts with spatial skill development in DHH and hearing people, exploring the range and heterogeneity of experiences with language and spatial skills.

Overall, this study was designed to explore individual differences in a variety of spatial factors and how they related to important STEM outcomes like spatial skills and interest in STEM. We explored anxiety about math, science, and spatial skills, spatial habits and visualization, experience with spatial language (ASL), and hearing status, investigating which of these factors would account for the most variance in spatial skills and interest in studying STEM fields. Although some of the purpose and design of the study was to investigate these factors in a sample of people who all had some spatial language experience, we did not find that self-reported ASL language experience had robust effects related to our outcome measures. Indeed, our results suggest that compared to other spatial factors, math and science anxiety were the most robust factors predicting spatial skills in mental rotation, and interest in studying STEM. As math and science anxiety increased, participants showed increased commission error rate on the MR task and decreased interest in studying STEM fields. When we consider important factors that could limit participation in STEM fields, especially for DHH individuals, math and science anxiety must be addressed as a priority. Future directions could explore math and science anxiety in DDBHH communities by designing accessible tasks, and how these anxieties affect performance on math and other spatial tasks, interest and success in STEM outcomes like classes, majors and career choices. In addition, future directions should also explore intervention strategies that would be efficacious at reducing the deleterious impact of anxiety on STEM outcomes. By designing accessible interventions for math anxiety and science anxiety that center the experiences of DHH students, we can develop intervention strategies that benefit the social and emotional experiences of all students, encouraging them to reach their potential and thrive in STEM.

## Methods

### Participants

**Fig. 1:**
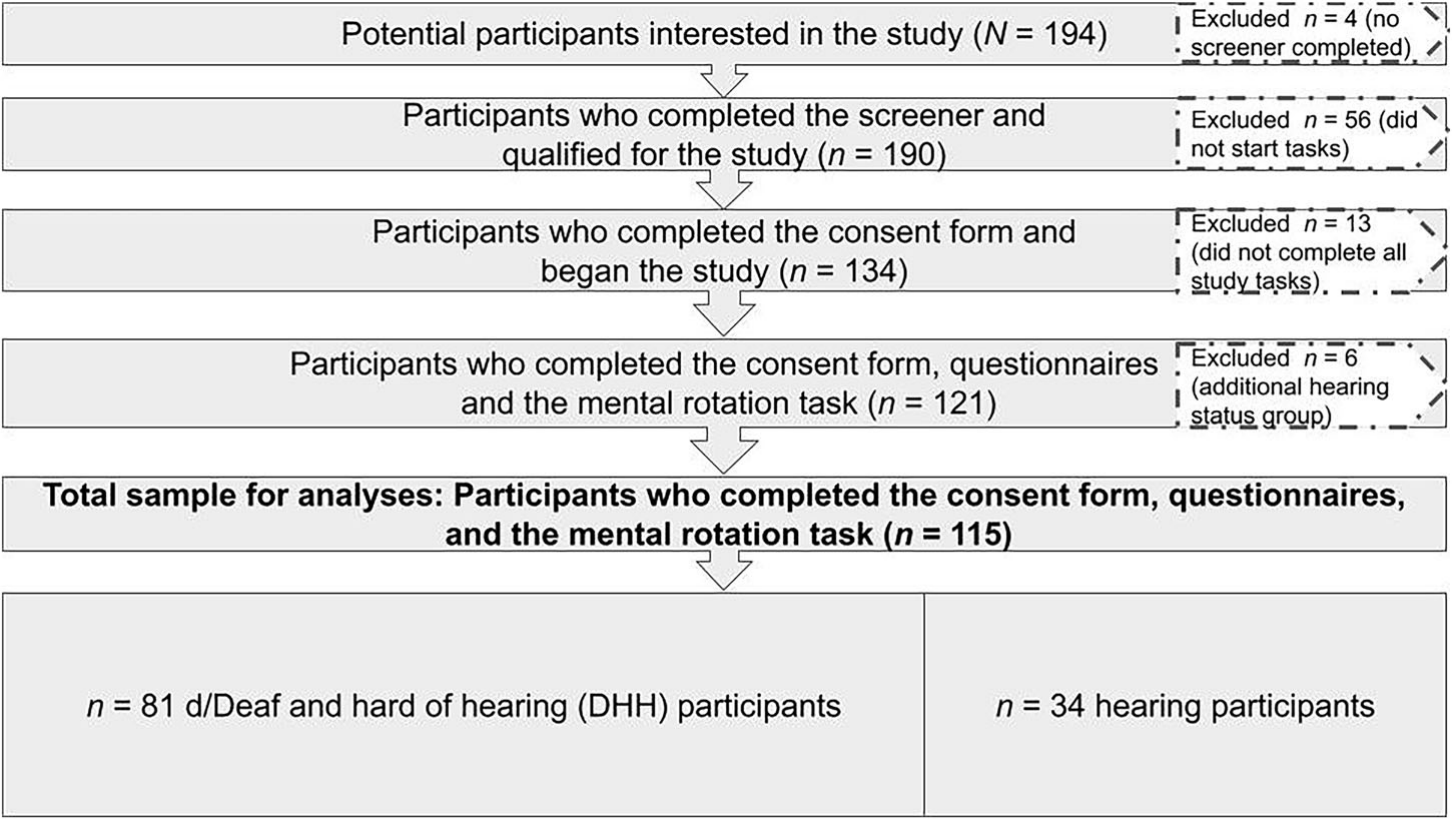
Participant sample size and inclusion criteria. Figure 1 showing the relative sample size and excluded participants from recruitment to the final sample size used for analyses. The final sample size included *n* = 115 participants, including *n* = 81 DHH participants and *n* = 34 hearing participants.

Participants were recruited to participate in an online study. Participants were recruited on the basis of having experience with a signed language (ASL), but were not recruited based on hearing status. Participants were screened to be between the ages of 18 and 60, be able to read and write in English, and have access to a computer in order to take the study. Participants were recruited through flyers, emails, and personal connections from signing communities in the Mid-Atlantic and Northeastern United States. Overall, 194 people contacted the lab who were interested in participating, *n* = 190 people completed the screening procedure. Of the participants who completed the consent form and began the study (*n* = 134), 13 participants did not complete both the survey responses and the mental rotation task (described below) and therefore were not included in the final sample (*n* = 121, Fig. 1).

This group of participants represents a diverse community of various language backgrounds, and a variety of hearing statuses. Participants were asked to “choose the option that best describes your hearing status.” Approximately half of our participants were d/Deaf (49.6%), 17.4% were hard of hearing, 28.1% were hearing, 2.5% were DeafBlind, and 2.5% chose to specify their status by writing in a response (most responses included another disability such as autism or auditory processing disorder). For this study, we made the decision not to include the data from the DeafBlind and self-described participants (*n* = 6, most reported auditory processing disorder or late-deafened; Fig. 1), because their experiences were thought to be differentiated from the rest of the sample in their experiences of disability, and it did not seem representative to include these data together with the participants who reported being deaf and hard of hearing. Because we do not want to overgeneralize our results based on a small group of people to a very heterogeneous and diverse group, we have omitted their data from the analyses. For the purposes of analysis, we chose to combine participants who were deaf and hard of hearing (DHH) into one group, and hearing into another group. Although there are many diverse experiences of language, access to sound, and cultural backgrounds across both groups, here we chose to explore the experiences of DHH people (*n* = 81) together contrasted with the experiences of hearing people (*n* = 34). Our sample of DHH/hearing groups for analysis had *n* = 115 participants.

Participants in this group of DHH and hearing participants had a wide range of experience with American Sign Language (ASL), although all participants reported having at least some experience with the language in order to qualify for study participation. The largest share of participants (25.2%) learned ASL as infants (age birth-2 years), 7.8% learned ASL as toddlers (age 2–4 years), 16.5% learned in young childhood (age 4–10 years), 5.2% learned as pre-teens (age 10–13 years), 16.5% learned as adolescents/high school-aged children (age 14–17 years), 18.3% learned as young adults/college-aged (age 18–22 years), 2.6% learned ASL as young adults/post college-aged (age 23–26 years), 7.0% learned ASL as adults (age 26+ years), and.8% of participants reported N/A or chose to write in a response. Of the *n* = 58 participants who reported the number of years of ASL experience, participants reported between 1 and 48 years of experience with ASL, with an average of 18.9 years of experience (SD: 12.53 years). Overall, participants in this study had a wide variety of experiences with ASL.

Participants were between the ages of 18 and 57 (*M* = 29.73, *SD* = 9.43). Participants self-reported gender: 26% “Male” (*n* = 31), 60% “Female” (*n* = 72), 10% “Nonbinary” (*n* = 12), 4% chose to self-describe (*n* = 5) and 0.8% chose not to share this information (*n* = 1). Participants self-reported race (participants could select more than one option): 75.2% White, 4.1% Black or African American, 2.5% American Indian or Alaskan Native, 8.3% Asian, 0.8% Native Hawaiian or Pacific Islander, 2.5% Chose to self-describe, and 3.3% chose not to share this information. The majority of participants did not identify as Latino/a/x (85.1%); 12.4% were Latino/a/x, and 2.5% did not provide this information.

### Procedure

Participants in this study were recruited to participate in an online study which would take approximately 1 hour to complete. Interested potential participants completed a brief online eligibility screener form, including a question assessing a minimum amount of experience with ASL. Eligible participants were sent a link to the online study. All participants completed an online consent form at the start of the study, and a video ASL version of this consent form was provided in tandem with the English form. This study was reviewed and approved by the Gallaudet University Institutional Review Board (IRB-FY21-13).

Participants completed three tasks in a counterbalanced order: a mental rotation task, an arithmetic task, and self-report demographics and questionnaires. More information about the mental rotation task and questionnaires is provided in subsequent sections. Unfortunately, due to a coding error, the behavioral data from the arithmetic task were not usable and will not be discussed in this manuscript. We requested that all participants complete all tasks on a laptop or desktop computer and not on a tablet or phone, which would affect the appearance and presentation of different stimuli. After completing the online tasks, participants were compensated for their time with an online gift card.

### Mental Rotation (MR) Task

To assess spatial skills, participants completed a short, online assessment of their mental rotation abilities^19,60,61^. Each trial displayed two two-dimensional diagrams side by side. The diagrams were similar sets of 10 3-dimensional white blocks on a gray background (all images 720 × 720 pixels). The task required a decision whether either set of blocks could be rotated so that the two diagrams would show identical pictures or a mirror image. Half of the trials presented were “SAME” trials where the rotated figures were identical, and in the other half, the rotated figures were mirror images where the correct response was “DIFFERENT.” Participants completed seven practice trials with feedback and then completed 36 trials. The response period was seven seconds during each trial before the program automatically advanced, which was judged to make the task appropriately difficult during piloting. At random intervals, five self-timed breaks appeared during the task to encourage participants to pay attention and respond during the task itself. Each break required a response to advance the task, ensuring that the task would not advance all the way through without any participant responses. The online survey platform recorded the accuracy and response times of responses. However, due to variability in web browser and internet connection speed, here we will focus only on the accuracy of responses out of 36 total trials.

Because the task automatically advanced after a timeframe of 7 seconds, it was possible for participants to perform below chance level if they did not provide a response before the time limit. On average, participants had 2.2 omission errors, or errors where they provided no response during the 36 trials (SD = 2.54, average omission error rate = 6.04%, SD = 7.06%). For the purposes of our analyses, we chose to focus on commission error rate, or the proportion of incorrect responses compared to the total number of responses made. On average, participants made 10.88 commission errors (wrong responses; SD = 4.90), and had a mean commission error rate of 31.43% (SD = 14.4%).

### Self-reported demographics and questionnaires

In this section, participants responded to a variety of different questions about themselves, including demographic information and questionnaires about academic anxieties, experiences, and performance. Questionnaires were presented in a randomized order for each participant. All questionnaires included optional short videos of the instructions in ASL, and some questionnaires included videos of individual questionnaire items that could be viewed in ASL. All videos were signed by a fluent deaf signer who had native proficiency in ASL and English. Information about featured questionnaires is provided in Table 4.

**Table 4:** Self-report questionnaires assessing emotions and performance related to academics

Questionnaire	Description	Example Items	*N*	Mean (SD)	Scale Range (Observed)
Math Anxiety: AAI-Math	Math Anxiety Subscale of AAI; Higher scores = more anxiety	“For some reason even though I study, math seems unusually hard for me.” “Figuring out mathematical problems does not appeal to me.”	121	2.71 (0.93)	1–5 Disagree-Agree (1–4.6)
Science Anxiety: AAI-Science	Science Anxiety Subscale of AAI; Higher scores = more anxiety	“I feel stressed trying to focus a microscope.” “Using a thermometer in order to record the boiling point of a heating solution makes me nervous.”	121	1.99 (0.53)	1–5 Disagree-Agree (1–3.3)
Spatial Anxiety: SAS-MM	Spatial Anxiety Scale, specifically Mental Manipulation subscale; Higher scores = more anxiety	“Asked to imagine and mentally rotate a 3-dimensional figure.” “Imagining on a test what a 3-dimensional landscape model would look like from a different point of view”	119	2.80 (0.93)	1–5 Rate Anxiety (1–5)
Spatial Habits: SHOMI-VIS	Spatial Habits of Mind Inventory, specifically Visualization subscale; Higher scores = more spatial habits	“When I am thinking about a complex idea, I use diagrams, maps, and/or graphics to help me understand.” “It is helpful for me to visualize physical phenomena such as hurricanes or weather fronts to understand them.”	121	3.68 (0.55)	1–5 Disagree-Agree (2.25–4.88)
Visualizer:VVQ-Vis	Visualizer-Verbalizer Questionnaire, specifically visualizer subscale; Lower scores = more visual tendencies	“I find illustrations or diagrams help me when I’m reading” “I like newspaper articles that have graphs”	121	1.26 (0.14)	1: True 2: False (1–1.8)
STEM Interest	Asked about interest in studying various different academic subjects; Average response of “Formal Sciences” and “Physical Sciences”	“What is your interest in the following areas of study?” “Formal Sciences (Mathematics, Computer Science)” “Physical Sciences (Biology, Physics, Chemistry)”	112	2.19 (0.95)	1-4 “Not at all interesting to me” -“Extremely interesting to me” (1–4)
Language Preference (ASL): *Lang-Pref-ASL	Asked about communication preferences for ASL receptive and expressive skills; Higher scores = stronger preference for ASL	“Indicate your preference for using each method [ASL] YOURSELF to communicate with others” “Indicate your preference FOR OTHERS to use each method of communication [ASL] to communicate with you”	120	34.76 (22.08)	−50 to +50 (−50 to +50)
*ASL-Comfort	Asked about comfort with ASL expressive and receptive skills and role in identity; Higher scores = more comfort with ASL	“I feel very comfortable understanding others when they use ASL to communicate with me.” “My ability to communicate using ASL is very important to my identity.”	121	4.27 (.94)	1–5 Disagree-Agree (1–5)
*ASL-Age	Asked about ASL age of Acquisition--reverse scored so that earlier AoA is scored as a larger number; Higher scores = early age of acquisition	“What age did you start learning ASL?“	121	5.19 (2.27)	0–8 Late – Early (0–8)
ASL-Years	Asked about number of years using ASL; Larger scores = Larger number of years using ASL	“How many years have you been regularly using ASL to communicate?“	58	18.98 (12.38)	Raw Number of Years (1–48)
ASL Experience: ASL-Summary	Average Z-Score of *ASL Language Variables; Larger scores = More comfort and experience using ASL	See above Lang-Pref-ASL, ASL-Comfort, and ASL-Age	120	0.0027 (0.88)	Average Z-Score (–2.99 – 0.90)

Table summarizing questionnaires and variables of interest. Mean scores represent the average response score (which falls within the specified range). Example items are provided for each scale/subscale. The ASL Experience construct measured by the ASL-Summary variable represents the average of each participant’s z-scored ASL variables: ASL Language Preference, ASL Comfort, ASL Age of Acquisition. ASL-Years was not used in this summary calculation because this variable was only completed by less than half of the included participants. An asterisk indicates measures that were combined to create the ASL-Summary score to indicate ASL Experience.

### Math, science, and spatial anxiety

Math anxiety and Science Anxiety were assessed with the Academic Anxiety Inventory (AAI^47^). This 50-item questionnaire includes 10-item subscales to assess separable domains of academic anxiety, including math, science, writing, test, and trait anxiety. This questionnaire was optimized to explore anxiety related to both math anxiety and science anxiety. Participants also completed the MARS^62,63^, but the researchers chose to utilize the AAI-Math subscale for the present analyses because it had a parallel structure to the other AAI subscales, such as AAI-Science. AAI-Math and the MARS were highly correlated with one another, *r*(119) = 0.63, *p* < 0.001.

To assess anxiety about spatial cognition, participants completed the Spatial Anxiety Scale (SAS^45^). This questionnaire was designed to assess anxiety about three specific domains of spatial skills: spatial navigation, spatial imagery, and mental manipulation. Here, we focus specifically on the mental manipulation subscale (SAS-MM), as this subscale represents the ability to hold and control spatial information. These skills are likely most germane to skills utilized in a mental rotation task.

### Spatial habits

Participants also completed two questionnaires related to spatial habits and spatial thinking. Participants completed the Spatial Habits of Mind Inventory (SHOMI^18^). This questionnaire assessed the extent to which participants regularly engage in various types of spatial thinking, such as pattern recognition, spatial description, visualization, spatial concept use, and spatial tool use. The visualization subscale was thought to be most related to mental rotation and spatial language skills, so this subscale was used for analyses. Similarly, participants also completed the Visualizer-Verbalizer Questionnaire (VVQ^58,59,64^, and the visualizer subscale (VVQ-Vis) was the focus of our analyses. This questionnaire measures the extent to which individuals have preferences about using visual or verbal skills on a regular basis. Here we use the term “spatial habits” to refer to scores across the SHOMI and The VVQ-Vis, in which we assessed participants’ self-reported habitual use and aptitude in using different spatial skills and behaviors that rely on spatial cognition.

### American sign language (ASL) experience

Participants responded to a variety of questions asking about their language background and experience. Questions were designed to assess comfort, extent of experience, age of acquisition, skill level, and importance to a sense of personal identity. Participants made ratings about both their receptive and expressive skills in ASL, as well as other language skills including spoken and written English, Signed Exact English (SEE), and were also given an option to make similar ratings of another language. As ASL was the spatial language of interest for this study, we will only focus on ASL as the language variable for the purposes of these analyses. To create one variable that summarized a variety of experiences with ASL, we chose to combine these ratings by Z-scoring each subscale (these are represented with * in Table 4), and then taking an average of the Z-scores within each participant. Average scores for our “ASL Summary” range from −2.99 to 0.9, indicating a range of low to high ASL preferences, skill levels, and comfort with ASL.

In addition to the questionnaires listed in Table 4, participants also completed additional questionnaires that were less relevant to the current analysis, such as the Emotion Regulation Questionnaire (ERQ^65^), and additional questions about math attitudes and experience^66^.

## Supplementary information

Supplementary Material

## Data Availability

Due to the small nature of the DDBHH communities, the researchers are not able to publicly share all behavioral data collected to protect participant confidentiality and privacy, as multiple pieces of demographic information can be used to triangulate individual participants’ data. Only deidentified compiled data with demographic information removed will be available for sharing through the Open Science Framework. Supplementary materials, an appendix of materials, and preprint versions of this manuscript are available through the Open Science Framework through PsyArXiv: https://osf.io/c83t6/files/osfstorage Researchers should contact the corresponding authors to request access to raw data or data that includes demographic information.

## Code availability

The R code used for analysis will also be available through the Open Science Framework (https://osf.io/c83t6/files/osfstorage).
