# Supplementary material for: Math anxiety and science anxiety are associated with spatial cognition and STEM interest in deaf, hard of hearing, and hearing people
Source: NPJ Sci Learn. 2025 Jul 9;10:45. doi: 10.1038/s41539-025-00336-z (PMC12238523; doi:10.1038/s41539-025-00336-z)
Supplement: Supplementary file 1 — Supplementary Material [file 41539_2025_336_MOESM1_ESM.pdf]

Supplementary Materials

RESULTS

Supplementary Results 1: Gender Analyses

Analysis Plan

To evaluate differences in gender, participants were grouped into three categories: male, female, and participants who reported “nonbinary” and “self-described” were combined into a third category, referred to as “nonbinary/self-described” (NBSD) for our analyses. One participant who chose not to share gender-related information was excluded from these analyses. We used one way ANOVAs and post-hoc t-tests with a Tukey method for comparing three estimates to evaluate the differences between genders (evaluated as 3 self-reported groups: male, female, nonbinary/self-described, NBSD).

Supplementary Table 1. Gender comparisons with Academic Anxiety, Spatial Habits and Skills, ASL Experience, and STEM Interest.

	Math Anxiety	***Science Anxiety***	***Spatial Anxiety***	***Spatial Habits of Mind***	***VVQ-Visualizer***	***ASL-Summary***	***MR Accuracy***	***STEM Interest***
Gender Differences	$F(2,111) = 3.16$, $p = .04$*	$F(2,111) = 7.38$, $p < .001$***	$F(2,109) = 5.41$, $p = .006$**	$F(2,111) = 2.71$, $p = .07$	$F(2,111) = 3.32$, $p = .04$*	$F(2,110) = .44$, $p = .64$	$F(2,111) = 3.00$, $p = .05$	$F(2,109) = 8.55$, $p < .001$***

Note. One-way ANOVAs for gender analyses were calculated with alpha = .05. The directions for statistically significant effects for differences gender are discussed in-text, below. * < .05, ** < .01, *** < .001.

Did we observe gender differences in anxiety, spatial skills, and STEM interests? Because of pervasive social stereotypes suggesting that men may have an advantage in studying STEM topics, previous research has suggested that women may report increased math anxiety and spatial anxiety (Sokolowski et al., 2018). We evaluated differences in gender using three groups: males, females, and nonbinary/self-described gender. In our hypotheses, we predicted that men would report decreased anxiety, and increased spatial habits and skills compared to women. We did not have specific hypotheses pertaining to the NBSD group, as this group is heterogeneous and comparatively smaller, so we hesitate to interpret or generalize these results. Here, we explored whether we would observe gender differences across math, science, and spatial anxiety, spatial habits, and differences in outcome measures such as mental rotation accuracy, and interest in STEM (Supplementary Table 1).

In this sample, we found gender differences in math, science, and spatial anxiety. In math anxiety, males ($M = 2.37$, $SE = .17$) reported significantly less math anxiety than females ($M = 2.86$, $SE = .11$), $t(111) = 2.49$, $p = .04$, but neither group significantly differed from NBSD individuals ($M = 2.81$, $SE = .25$). For science anxiety, males ($M = 1.70$, $SE = .09$) reported significantly less science anxiety than females ($M = 2.06$, $SE = .06$), $t(111) = -3.13$, $p = .006$, and significantly less science anxiety than the NBSD group ($M = 2.25$, $SE = .13$), $t(111) = -3.42$, $p = .003$. Women and the NBSD group did not report significantly different levels of science anxiety, $p > .05$. For spatial anxiety, men reported significantly decreased spatial anxiety ($M = 2.36$, $SE = .16$), compared to women ($M = 2.92$, $SE = .11$), $t(111) = -2.89$, $p = .01$, and NBSD people ($M = 3.14$, $SE = .24$), $t(111) = -2.72$, $p = .02$. Women and NBSD people did not significantly differ in their self-reported levels of spatial anxiety, $p > .05$.

We also explored gender differences in spatial habits and spatial skills (Supplementary Table 1). We did not observe gender differences in spatial habits, $p > .05$. However, we did observe gender differences in tendency to use visualization or be a "visualizer," such that males ($M = 1.21$, $SE = .02$) reported a stronger tendency and preference for using visuospatial skills compared to the NBSD group ($M = 1.33$, $SE = .04$), $t(111) = -2.56$, $p = .04$. The females ($M = 1.26$, $SE = .02$) did not significantly differ from either group, *p*s > .05. We did not observe any differences between genders in self-reported experience and expertise with a spatial language (ASL-Summary), $p > .05$. We predicted that males would show increased accuracy in the MR task, and this was not supported by our results, which showed no significant differences in MR accuracy based on gender, $p > .05$.

Finally, we explored whether gender would be associated with differences in self-reported interest in studying STEM fields. We predicted that males would report more interest in studying STEM than females. Indeed, this prediction was supported by our results (Supplementary Table 1). Males ($M = 2.73$, $SE = .16$) self-reported significantly higher interested in studying STEM fields compared to females ($M = 1.93$, $SE = .11$), $t(109) = 4.13$, $p = .0002$. The NBSD group ($M = 2.25$, $SE = .24$) did not significantly differ from males or females, *p*s > .05.

Taken together, these gender comparisons suggest that our results show increased anxiety associated with quantitative content and decreased interest in studying STEM for females. This is consistent with much of the previous literature suggesting increased anxiety and decreased motivation for females compared to males. However, our results suggest that males and females did not show robust differences in spatial thinking or habits, and they did not differ in actual spatial task performance.

Linear Regressions Including Gender

Mental Rotation

Supplementary Table 2. Regression results using Mental Rotation Accuracy as the criterion

Predictor	*b*	*b* 95% CI [LL, UL]	sr^2	sr^2 95% CI [LL, UL]	Fit
(Intercept)	23.51**	[21.53, 25.48]			
Spatial Anxiety (SAS-MM)	-0.26	[-1.25, 0.73]	.00	[-.01, .02]	
Math Anxiety (AAI-Math)	-1.67**	[-2.71, -0.63]	.08	[-.01, .17]	
Science Anxiety (AAI-Science)	-1.11*	[-2.16, -0.05]	.03	[-.03, .09]	
Spatial Habits of Mind (SHOMI-Vis)	0.04	[-1.08, 1.15]	.00	[-.00, .00]	
VVQ-Visualizer	0.19	[-0.83, 1.21]	.00	[-.01, .01]	
ASL-Summary	-0.13	[-1.38, 1.12]	.00	[-.01, .01]	
Hearing Status	-0.02	[-2.45, 2.42]	.00	[-.00, .00]	
Gender (M vs. F)	-1.00	[-3.30, 1.30]	.01	[-.02, .03]	
Gender (M vs. NBSD)	0.31	[-3.08, 3.71]	.00	[-.01, .01]	

					Fit
					R^2 = .227** 95% CI[.04,.30]

Note. A significant *b*-weight indicates the semi-partial correlation is also significant. *b* represents unstandardized regression weights. sr^2 represents the semi-partial correlation squared. *LL* and *UL* indicate the lower and upper limits of a confidence interval, respectively. Math and Science Anxiety are significant predictors of Mental Rotation Accuracy, as in the model reported in the main manuscript. The contrasts with Gender are not significant.
* indicates p < .05. ** indicates p < .01.

Supplementary Table 3. Regression results using STEM Interest as the criterion

Predictor	*b*	*b* 95% CI [LL, UL]	sr^2	sr^2 95% CI [LL, UL]	Fit
(Intercept)	2.72**	[1.96, 3.48]			
Spatial Anxiety (SAS-MM)	-0.10	[-0.25, 0.05]	.01	[-.02, .03]	
Math Anxiety (AAI-Math)	-0.44**	[-0.60, -0.28]	.15	[.05, .24]	
Science Anxiety (AAI-Science)	-0.17*	[-0.34, -0.01]	.02	[-.02, .06]	
Spatial Habits of Mind (SHOMI-Vis)	0.10	[-0.07, 0.26]	.01	[-.01, .03]	
VVQ-Visualizer	0.02	[-0.13, 0.17]	.00	[-.00, .01]	
ASL-Summary	-0.14	[-0.33, 0.05]	.01	[-.02, .04]	
Hearing Status	0.01	[-0.35, 0.38]	.00	[-.00, .00]	
Mental Rotation Accuracy	-0.01	[-0.04, 0.02]	.00	[-.01, .02]	
Gender (M vs. F)	-0.41*	[-0.76, -0.07]	.03	[-.02, .07]	
Gender (M vs. NBSD)	-0.01	[-0.52, 0.50]	.00	[-.00, .00]	
					R^2 = .498** 95% CI[.30,.56]

Note. A significant *b*-weight indicates the semi-partial correlation is also significant. *b* represents unstandardized regression weights. sr^2 represents the semi-partial correlation squared. *LL* and *UL* indicate the lower and upper limits of a confidence interval, respectively. Math and Science Anxiety are significant predictors of STEM Interest, as in the model reported in the main manuscript. The contrast between Male and Female participants is significant--males reported more STEM interest than females.
* indicates p < .05. ** indicates p < .01.

Past research has suggested that there may be gender differences in self-reported math anxiety or performance of visuospatial skills. Our results suggested that female participants reported increased math, science, and spatial anxiety compared to male participants. In addition, female participants reported reduced interest in studying STEM compared to male participants. However, the groups did not significantly different in actual performance of visuospatial skills. These results suggest that participants may reflect widespread stereotypes about women underperforming in STEM, but that these attitudes and anxieties do not reflect any actual differences in ability. However, this study was not optimized to study differences in gender. As a result, we made the decision not to include self-reported gender in the main multiple regression analyses, and instead these analyses are elaborated in the supplementary material. Our participant sample included more female participants than male participants, meaning that many of our comparisons are not optimized to compare differences in gender. In addition, our gender analyses included a smaller group of people who reported non-binary, self-described gender identities. Because this is a heterogeneous and diverse group, and because our design was not well-balanced to compare female, male, and NBSD groups, we hesitate to draw strong conclusions related to gender differences in outcomes related to STEM in this study. The present study represents a small step forward in disambiguating some of the effects related to visuospatial skills and STEM outcomes, and further, larger scale research with larger sample sizes will be needed to establish more broadly applicable theories about these factors.

Supplementary Results 2: Accuracy Analyses

Our accuracy measure represents the total number of correct responses out of 36 total trials in the Mental Rotation task. The analyses in the main manuscript focused on commission errors, or errors where an incorrect response was made. Here our accuracy measure includes both commission and omission errors, or errors where no response was made.

Supplementary Table 4. Zero-Order Correlations between Academic Anxiety, Spatial Habits and Skills, ASL Experience, STEM Interest, and Differences in Hearing Status and Gender.

	Math Anxiety	***Science Anxiety***	***Spatial Anxiety***	***Spatial Habits of Mind***	***VVQ-Vis***	***ASL-Summary***	***MR Accuracy***	***STEM Interest***
Math Anxiety	--	.25*	.26*	-.42***	.16	.00	-.34**	-.53***
Science Anxiety	.26**	--	.30**	-.30**	.30**	-.13	-.27*	-.36**
Spatial Anxiety	.28**	.29**	--	-.09	.16	.05	-.15	-.32**
Spatial Habits of Mind	-.44***	-.31***	-.21*	--	-.34**	.00	.23*	.42***
VVQ-Vis	.19*	.27**	.18	-.40***	--	-.17	-.10	-.14
ASL-Summary	.12	.05	.06	-.05	-.08	--	.10	-.07
MR Accuracy	-.40***	-.33***	-.21*	.25**	-.10	-.08	--	.21
STEM Interest	-.61***	-.37***	-.33***	.41***	-.16	-.19*	.27**	--

Hearing Status	*t*(56.67) = .68, *p* = .50 NS	*t*(75.18) = 2.02, *p* = .04* DHH > H	*t*(56.68) = 1.11, *p* = .27 NS	*t*(61.48) = -1.19, *p* = .23 NS	*t*(67.51) = .07, *p* = .94 NS	*t*(41.62) = 5.74, *p* < .001*** DHH > H	*t*(70.19) = -.90, *p* = .37 NS	*t*(59.74) = -1.30, *p* = .20 NS

Note. Correlations below the diagonal represent relations in all participants (*N* = 115). Correlations above the diagonal (in gray) were calculated in DHH participants only (*N* = 81). Pearson correlations and t-tests for hearing status were calculated with alpha = .05. Statistically significant effects for differences in hearing status and gender are discussed in-text below. DHH = deaf and hard of hearing, H = Hearing, NS= no significant group differences.* < .05, ** < .01, *** < .001.

Supplementary Table 5. Regression results using Mental Rotation Accuracy as the criterion

Predictor	*b*	*b* 95% CI [LL, UL]	sr^2	sr^2 95% CI [LL, UL]
(Intercept)	23.00**	[21.85, 24.15]		
Spatial Anxiety (SAS-MM)	-0.30	[-1.27, 0.66]	.00	[-.01, .02]
Math Anxiety (AAI-Math)	-1.70**	[-2.74, -0.67]	.08	[-.01, .17]
Science Anxiety (AAI-Science)	-1.15*	[-2.16, -0.14]	.04	[-.03, .10]
Spatial Habits of Mind (SHOMI-Vis)	0.15	[-0.94, 1.24]	.00	[-.01, .01]
VVQ-Visualizer	0.26	[-0.73, 1.26]	.00	[-.01, .02]
ASL-Summary	-0.11	[-1.36, 1.13]	.00	[-.00, .01]
Hearing Status	-0.21	[-2.62, 2.19]	.00	[-.00, .01]

Overall Fit: R^2 = .217**, 95% CI [.05,.30]

Note. Spatial Anxiety, Math Anxiety, Science Anxiety, Spatial Habits of Mind, and the VVQ-Visualizer subscale were all Z-scored for this analysis. A significant *b*-weight indicates the semi-partial correlation is also significant. *b* represents unstandardized regression weights (though, on the z-scored variables, these represent standardized scores). sr^2 represents the semi-partial correlation squared. *LL* and *UL* indicate the lower and upper limits of a confidence interval, respectively. * indicates p < .05. ** indicates p < .01.

Supplementary Table 6. Regression results using STEM Interest as the criterion

Predictor	*b*	*b* 95% CI [LL, UL]	sr^2	sr^2 95% CI [LL, UL]
(Intercept)	2.40**	[1.67, 3.12]		
Spatial Anxiety (SAS-MM)	-0.12	[-0.27, 0.03]	.01	[-.02, .05]
Math Anxiety (AAI-Math)	-0.45**	[-0.62, -0.28]	.15	[.05, .25]
Science Anxiety (AAI-Science)	-0.20*	[-0.35, -0.04]	.03	[-.02, .08]
Spatial Habits of Mind (SHOMI-VIS)	0.13	[-0.04, 0.30]	.01	[-.02, .05]

VVQ-Visualizer	0.04	[-0.11, 0.20]	.00	[-.01, .01]
ASL-Summary	-0.13	[-0.33, 0.06]	.01	[-.02, .04]
Hearing Status	-0.06	[-0.43, 0.31]	.00	[-.01, .01]
Mental Rotation Accuracy	-0.01	[-0.04, 0.02]	.00	[-.01, .01]

Fit: R^2 = .459**, 95% CI[.27,.53]

Note. Spatial Anxiety, Math Anxiety, Science Anxiety, Spatial Habits of Mind, and the VVQ-Visualizer subscale were all Z-scored for this analysis. A significant *b*-weight indicates the semi-partial correlation is also significant. *b* represents unstandardized regression weights (though, on the z-scored variables, these represent standardized scores). sr^2 represents the semi-partial correlation squared. *LL* and *UL* indicate the lower and upper limits of a confidence interval, respectively.
* indicates p < .05. ** indicates p < .01. *** indicates p < .001.

Supplementary Results 3: Analyses considering DHH and Hearing Groups Separately

Spatial Skills: Which factor is the strongest predictor of performance on spatial skills?

For the DHH Group:

Supplementary Table 7. Regression results using MR Commission Error Rate as the criterion for the DHH Group (N = 80)

Predictor	*b*	*b* 95% CI [LL, UL]	*beta*	*beta* 95% CI [LL, UL]	sr^2	sr^2 95% CI [LL, UL]	*r*
(Intercept)	33.80**	[30.04, 37.56]					
Spatial Anxiety (SAS-MM)	0.49	[-3.07, 4.05]	0.03	[-0.20, 0.27]	.00	[-.01, .01]	.14
Math Anxiety (AAI-Math)	3.56	[-0.20, 7.33]	0.23	[-0.01, 0.48]	.04	[-.04, .12]	.32**
Science Anxiety (AAI-Science)	2.65	[-0.90, 6.20]	0.18	[-0.06, 0.42]	.03	[-.04, .09]	.27*
Spatial Habits of Mind (SHOMI-VIS)	-1.51	[-5.33, 2.31]	-0.10	[-0.35, 0.15]	.01	[-.03, .04]	-.23*
VVQ-Visualizer	-0.72	[-4.17, 2.73]	-0.05	[-0.29, 0.19]	.00	[-.02, .02]	.09
ASL-Summary	-3.09	[-9.01, 2.83]	-0.12	[-0.34, 0.11]	.01	[-.03, .06]	-.13

R^2 = .162*
95% CI[.00,.26]

Note. A significant *b*-weight indicates the beta-weight and semi-partial correlation are also significant. *b* represents unstandardized regression weights. *beta* indicates the standardized regression weights. sr^2 represents the semi-partial correlation squared. *r* represents the zero-order correlation. *LL* and *UL* indicate the lower and upper limits of a confidence interval, respectively.
* indicates *p* < .05. ** indicates *p* < .01.

Hearing Group:

Supplementary Table 8. Regression results using MR Commission Error Rate as the criterion for the Hearing Group (N = 34)

Predictor	*b*	*b* 95% CI [LL, UL]	*beta*	*beta* 95% CI [LL, UL]	sr^2	sr^2 95% CI [LL, UL]	*r*
(Intercept)	33.53**	[28.88, 38.18]					
Spatial Anxiety (SAS-MM)	3.03	[-0.91, 6.97]	0.24	[-0.07, 0.56]	.05	[-.06, .15]	.39*
Math Anxiety (AAI-Math)	5.45*	[1.38, 9.52]	0.46	[0.12, 0.80]	.14	[-.03, .31]	.58**
Science Anxiety (AAI-Science)	5.11*	[0.67, 9.55]	0.34	[0.04, 0.64]	.10	[-.05, .25]	.49**
Spatial Habits of Mind (SHOMI-VIS)	2.05	[-3.13, 7.23]	0.16	[-0.25, 0.57]	.01	[-.04, .06]	-.30
VVQ-Visualizer	0.61	[-4.03, 5.25]	0.04	[-0.30, 0.39]	.00	[-.02, .02]	.20
ASL-Summary	1.41	[-2.24, 5.06]	0.11	[-0.18, 0.41]	.01	[-.04, .06]	.23

R2 = .518**
95% CI[.11,.62]

Note. A significant *b*-weight indicates the beta-weight and semi-partial correlation are also significant. *b* represents unstandardized regression weights. *beta* indicates the standardized regression weights. sr^2 represents the semi-partial correlation squared. *r* represents the zero-order correlation. *LL* and *UL* indicate the lower and upper limits of a confidence interval, respectively.
* indicates $p < .05$. ** indicates $p < .01$.

Across the DHH and hearing groups, the hearing group replicates the results of the overall sample, such that math anxiety and science anxiety are significantly related to MR commission error rates. In the DHH group, math anxiety does not quite reach the alpha criterion for statistical significance, $p = .06$, and all other predictors do not reach the alpha criterion.

Interest in STEM: Which factor is the strongest predictor of Interest in STEM?
In DHH Group:

Supplementary Table 9. Regression results using STEM Interest as the criterion in DHH Group (N = 80)

Predictor	*b*	*b* 95% CI [LL, UL]	*beta*	*beta* 95% CI [LL, UL]	sr^2	sr^2 95% CI [LL, UL]	*r*
(Intercept)	2.12**	[1.64, 2.60]					
Spatial Anxiety (SAS-MM)	-0.15	[-0.34, 0.04]	-0.16	[-0.36, 0.05]	.02	[-.03, .07]	-.32**
Math Anxiety	-0.35**	[-0.56, -0.14]	-0.37	[-0.59, -	.10	[-.01, .21]	-.53**

(AAI-Math)				0.15]			
Science Anxiety (AAI-Science)	-0.17	[-0.37, 0.02]	-0.19	[-0.40, 0.02]	.03	[-.03, .09]	-.35**
Spatial Habits of Mind (SHOMI-VIS)	0.21	[-0.00, 0.41]	0.22	[-0.00, 0.44]	.03	[-.03, .10]	.41**
VVQ-Visualizer	0.04	[-0.15, 0.22]	0.04	[-0.17, 0.25]	.00	[-.01, .01]	-.14
ASL-Summary	-0.12	[-0.44, 0.20]	-0.07	[-0.27, 0.12]	.00	[-.02, .03]	-.07
Mental Rotation Comm. Error Rate	0.00	[-0.01, 0.02]	0.03	[-0.17, 0.24]	.00	[-.01, .01]	-.18

R^2 = .392**
95% CI[.15,.49]

Note. A significant *b*-weight indicates the beta-weight and semi-partial correlation are also significant. *b* represents unstandardized regression weights. *beta* indicates the standardized regression weights. sr^2 represents the semi-partial correlation squared. *r* represents the zero-order correlation. *LL* and *UL* indicate the lower and upper limits of a confidence interval, respectively.
* indicates $p < .05$. ** indicates $p < .01$.

Supplementary Table 10. Regression results using STEM Interest as the criterion in the Hearing Group (N =34)

Predictor	*b*	*b* 95% CI [LL, UL]	*beta*	*beta* 95% CI [LL, UL]	sr^2	sr^2 95% CI [LL, UL]	*r*
(Intercept)	1.32**	[0.40, 2.25]					
Spatial Anxiety (SAS-MM)	-0.18	[-0.45, 0.08]	-0.19	[-0.47, 0.09]	.03	[-.04, .09]	-.33
Math Anxiety (AAI-Math)	-0.77**	[-1.07, -0.47]	-0.84	[-1.17, -0.51]	.36	[.13, .60]	-.75**
Science Anxiety (AAI-Science)	-0.36*	[-0.68, -0.04]	-0.31	[-0.59, -0.04]	.07	[-.04, .18]	-.39*
Spatial Habits of Mind (SHOMI-VIS)	-0.12	[-0.46, 0.22]	-0.12	[-0.47, 0.23]	.01	[-.02, .04]	.37*
VVQ-Visualizer	0.00	[-0.30, 0.31]	0.00	[-0.28, 0.29]	.00	[-.00, .00]	-.20
ASL-Summary	-0.11	[-0.35, 0.13]	-0.12	[-0.37, 0.13]	.01	[-.03, .05]	-.25
Mental Rotation Comm. Error Rate	0.03	[-0.00, 0.05]	0.33	[-0.01, 0.67]	.05	[-.04, .14]	-.38*

R^2 = .676**
95% CI[.30,.74]

Note. A significant *b*-weight indicates the beta-weight and semi-partial correlation are also significant. *b* represents unstandardized regression weights. *beta* indicates the standardized regression weights. sr^2 represents the semi-partial correlation squared. *r* represents the zero-order correlation. *LL* and *UL* indicate the lower and upper limits of a confidence interval, respectively.
* indicates $p < .05$. ** indicates $p < .01$.

Across the DHH and Hearing groups, math anxiety is significantly negatively related to interest in studying STEM, $p < .01$, replicating the results observed in the full sample. In the hearing sample, science anxiety is also a significant predictor of STEM interest, $p < .001$, but this was not replicated in the DHH sample and did not reach statistical significance.
